# *Streptomyces* sp. MUM273b: A mangrove‐derived potential source for antioxidant and UVB radiation protectants

**DOI:** 10.1002/mbo3.859

**Published:** 2019-06-14

**Authors:** Loh Teng‐Hern Tan, Camille Keisha Mahendra, Yoon‐Yen Yow, Kok‐Gan Chan, Tahir Mehmood Khan, Learn‐Han Lee, Bey‐Hing Goh

**Affiliations:** ^1^ Novel Bacteria and Drug Discovery Research Group Microbiome and Bioresource Research Strength Jeffrey Cheah School of Medicine and Health Sciences Monash University Malaysia Bandar Sunway Selangor Darul Ehsan Malaysia; ^2^ Biofunctional Molecule Exploratory (BMEX) Research Group School of Pharmacy Monash University Malaysia Bandar Sunway Selangor Darul Ehsan Malaysia; ^3^ Institute of Biomedical and Pharmaceutical Sciences Guangdong University of Technology Guangzhou PR China; ^4^ Department of Biological Sciences, School of Science and Technology Sunway University Bandar Sunway Selangor Malaysia; ^5^ International Genome Centre Jiangsu University Zhenjiang China; ^6^ Division of Genetics and Molecular Biology, Institute of Biological Sciences, Faculty of Science University of Malaya Kuala Lumpur Malaysia; ^7^ The Institute of Pharmaceutical Sciences University of Veterinary and Animal Sciences Lahore Pakistan

**Keywords:** antioxidant, cosmeceutical, mangrove, *Streptomyces*, UV‐protective

## Abstract

Microbial natural products serve as a good source for antioxidants. The mangrove‐derived *Streptomyces* bacteria have been evidenced to produce antioxidative compounds. This study reports the isolation of *Streptomyces* sp. MUM273b from mangrove soil that may serve as a promising source of antioxidants and UV‐protective agents. Identification and characterization methods determine that strain MUM273b belongs to the genus *Streptomyces*. The MUM273b extract exhibits antioxidant activities, including DPPH, ABTS, and superoxide radical scavenging activities and also metal‐chelating activity. The MUM273b extract was also shown to inhibit the production of malondialdehyde in metal‐induced lipid peroxidation. Strong correlation between the antioxidant activities and the total phenolic content of MUM273b extract was shown. In addition, MUM273b extract exhibited cytoprotective effect on the UVB‐induced cell death in HaCaT keratinocytes. Gas chromatography–mass spectrometry analysis detected phenolics, pyrrole, pyrazine, ester, and cyclic dipeptides in MUM273b extract. In summary, *Streptomyces* MUM273b extract portrays an exciting avenue for future antioxidative drugs and cosmeceuticals development.

## Graphical abstract

The mangrove‐derived Streptomyces bacteria known as MUM273b has been evidenced to produce antioxidative compounds. This study reports the isolation of *Streptomyces* sp. MUM273b from mangrove soil that may serve as a promising source of antioxidants

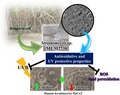

## 1 INTRODUCTION

Natural products refer to chemical compounds produced by living organisms, for instance, plants, animals, and microorganisms that benefit the producer (Katz & Baltz, 2016). Both terrestrial plants and microorganisms portray indispensable sources for bioactive natural products in drug discovery efforts particularly owing to their exceptionally capability in producing a great number of structurally diverse compounds (Cragg & Newman, 2001; Ma et al., 2018; Tan, Lee, et al., 2015; Tang et al., 2016). The natural products that exhibit pharmacological properties have been harnessed as clinically important drugs to treat diseases. Besides their direct therapeutic use, the fascinating molecular frameworks of natural products offer a range of diverse unique chemotypes as inspiration for the development of current clinically significant drugs or potential novel drugs (Chan, Tan, Chan, Lee, & Goh, 2016; Nielsen, 2002; Rodrigues, Reker, Schneider, & Schneider, 2016; Tan, Low, et al., 2019). Whereas the natural environment is regarded as a rich source of unique chemical diversity, the reduced trend in the discovery of new bioactive compounds as well as the frequent rediscovery of previously identified compounds have been an increasing challenge for the field of drug discovery from natural products (Lam, 2007; Li & Vederas, 2009). Despite that, many has turned their focus toward natural products derived from difficult‐to‐reach sources/habitats to increase the opportunities for finding novel chemical entities (Desbois, 2014; Xu, 2015).

As of today, estimated 50%–70% of all agents in clinical use are of natural product origin, majority of which are derived from bacteria, particularly the family *Actinomycetaceae* (Bérdy, 2012). Ever since the discovery of penicillin and followed by streptomycin as a result of systematic screening of soil actinomycetes by the Waksman (Schatz, Bugle, & Waksman, 1944), microorganisms have been exploited by humans for thousands of years as the biofactories to produce beneficial products for human (Katz & Baltz, 2016; Kemung et al., 2018). Microbes live in every kind of ecological niche, for example, in sediments, thermal vents, and also in extreme environments that are otherwise detrimental to most living organisms on earth (Ghosh, 2012). Previously, terrestrial microbes were known to be rich source of biologically active secondary metabolites with significant pharmaceutical or agrochemical applications. The discovery of various unique bioactive natural products from terrestrial‐derived microbes includes the antibiotics: penicillin, streptomycin, vancomycin; antitumor drugs: actinomycin and mitomycin; antiparasitic drug: avermectin, and immunosuppressant drug cyclosporin (Katz & Baltz, 2016). Given the issues of frequent rediscovery of previously identified compounds, many has paid great attention to microbes derived from unique or unusual ecological niches, including the mangrove ecosystem (Xu, 2015).

Mangroves are among the most‐prolific and biologically imperative ecosystems on earth, because they bring benefits to human society serving as important sources of food, medicines, fuel, and building materials (Walters et al., 2008) in addition to acting as natural barrier that protects shorelines from devastating natural forces (Alongi, 2008; Quarto, 2005). Besides, mangrove environments harbor high level of microbial diversity such as bacteria, fungi, cyanobacteria, microalgae, macroalgae, and protozoa. Great interest has been given to the exploitation of mangrove‐derived microbial natural products owing to their wide variety of bioactivities, which contribute immensely in the industrial and clinical applications (Demain & Sanchez, 2009; Gupta, Gigras, Mohapatra, Goswami, & Chauhan, 2003; Tan, Chan, et al., 2019). Particularly, the *Streptomyces* species as the largest genus of *Actinobacteria*, which has contributed enormously to mankind, has become a prolific producer for bioactive compounds with various bioactivities such as antioxidant, antimicrobial, antitumor, immunosuppressant, and neutroprotective properties (Dan & Sanawar, 2017; Kim et al., 2011; Law, Ser, Duangjai, et al., 2017; Law, Ser, Khan, et al., 2017; Ser et al., 2017; Tan, Chan, Lee, & Goh, 2016). Together with the numerous discovery of novel *Streptomyces* species with bioactive potentials recently such as *S. malaysiense* (Ser, Palanisamy, et al., 2016), *S. antioxidans* (Ser, Tan, et al., 2016), *S. humi* (Zainal et al., 2016), *S. colonosanans* (Law, Ser, Duangjai, et al., 2017), *S. euryhalinus* (Biswas, Choudhury, Mahansaria, Saha, & Mukherjee, 2017) from mangrove soil, mangrove‐derived *Streptomyces* represent irreplaceable resources in bioprospecting of natural products with potentially novel chemotypes and promising pharmacological properties.

UV radiation from sunlight has been known to induce harmful responses, including erythema, sunburn, and skin cancer (Brash et al., 1991). Among the three types of solar UV rays, UVB radiation can cause serious skin damage via DNA damage and/or production of reactive oxygen species (ROS) (Nishigori, Hattori, & Toyokuni, 2004). Antioxidants have been shown to exhibit protective effects against UV‐induced oxidative damage on skin cells (Salucci et al., 2014). Natural antioxidants are found abundantly in metabolites produced from the microbial world (Atanasova‐Penichon, Barreau, & Richard‐Forget, 2016; Dey, Chakraborty, Jain, Sharma, & Kuhad, 2016; Wang et al., 2017). Similarly, numerous mangrove‐derived strains of *Streptomyces* sp. with potential to produce antioxidants also have been reported previously (Law, Ser, Duangjai, et al., 2017; Law, Ser, Khan, et al., 2017; Sanjivkumar et al., 2016; Tan et al., 2017). Therefore, this study reports the isolation of a *Streptomyces* sp. MUM273b from Kuala Selangor mangrove soil, Malaysia. The antioxidant and protective potentials of *Streptomyces* sp. MUM273b extract against UVB‐induced cytotoxicity were evaluated. These findings were also well supported with the detection of potential antioxidative compounds by gas chromatography and mass spectrometry analysis. Taken together, this study has further instilled the notion that mangrove *Streptomyces* serves as a rich source of antioxidants which could greatly benefit future research to cope with oxidative damage induced by UV radiation on skin cells.

## 2 MATERIAL AND METHODS

### 2.1 Environmental sampling and strain MUM273b isolation

Mangrove soil samples were collected from Kuala Selangor, Malaysia. Specifically, the site of collection was denoted as MUM‐KS1 located at the coordinate (3° 21′ 45.8″ N 101° 18′ 4.5″ E). Prior to the soil sample collection, approximately 3 centimeter of top layer soil was removed. The soil sample was collected from the layer of 20‐centimeter depth and kept at −20°C before further processing. During the sample processing, the soil was air‐dried and finely grounded before subjected to wet heat pretreatment (15 min at 50°C) (Takahashi, Matsumoto, Seino, Iwai, & Omura, 1996). Serial dilution of the treated soil sample was performed with sterile water to 10–4. Diluted soil suspension was spread onto isolation medium International Streptomyces Project (ISP) 2 (Shirling & Gottlieb, 1966) which was added with cycloheximide (25 µg/ml) and nystatin (10 µg/ml). A 14 days incubation at 28°C was performed for the inoculated ISP2 agar plate. Pure colony of strain MUM273b was picked and purified with new ISP2 agar. Strain MUM273b was maintained on slants of ISP2 agar at room temperature for short‐term storage while in 20% (v/v) glycerol suspensions at −20°C for long‐term storage.

### 2.2 Identification of strain MUM273b by 16S rRNA phylogenetic analysis

**Figure 1:**
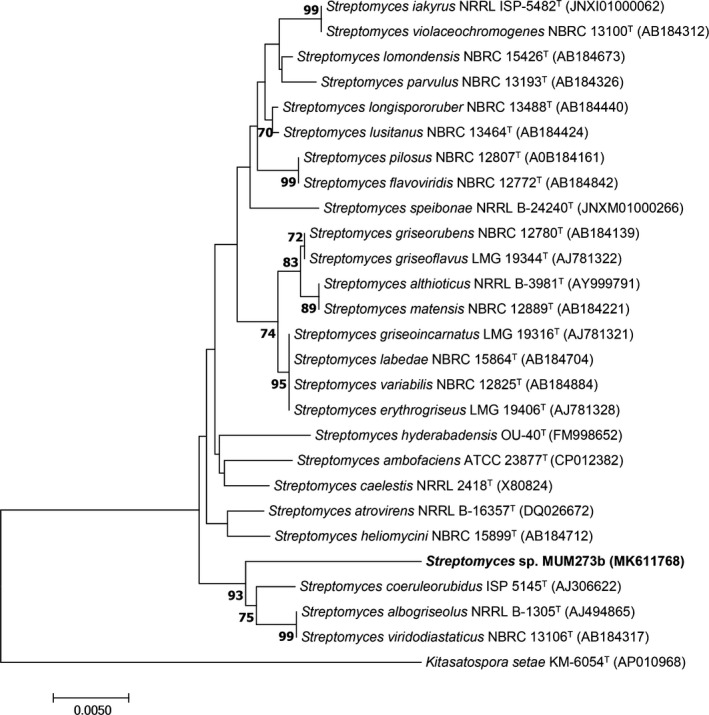
Neighbor‐joining phylogenetic tree based on the almost complete 16S rRNA sequences that shows the evolutionary relationships between the strain MUM273b (1406 bp) and representatives of some other related taxa. Bootstrap values (>50%) based on 1,000 re‐sampled datasets are shown at branch nodes. Bar, 0.005 substitutions per site

Genomic DNA of strain MUM273b was extracted and followed by 16S rRNA gene PCR amplification (Hong et al., 2009). The PCR amplification was conducted as described in Lee, Zainal, Azman, Eng, Ab Mutalib, et al. (2014), Lee, Zainal, Azman, Eng, Goh, et al. (2014) using the primer 27F (5′‐GTTTGATCCTGGCTCAG‐3′), 1492R (5′‐TACGGCTACCTTGTTACGACTT‐3′). The 16S rRNA gene of strain MUM273b was sequenced prior to alignment using the CLUSTAL‐X software. The representative gene sequences of related type strains of the genus *Streptomyces* were retrieved from the Genbank database (Thompson, Gibson, Plewniak, Jeanmougin, & Higgins, 1997). The alignments were checked and adjusted manually before proceeding to phylogenetic tree construction. The phylogenetic tree of strain MUM273b (Figure 1) was constructed with the neighbor‐joining algorithm (Saitou & Nei, 1987) using MEGA version 6.0 (Tamura, Stecher, Peterson, Filipski, & Kumar, 2013). Kimura's two‐parameter model was used to compute the evolutionary distances for the neighbor‐joining algorithm (Kimura, 1980). The sequence similarities were calculated based on EzTaxon‐e server (http://www.ezbiocloud.net/) (Kim et al., 2012). The bootstrapping based on 1,000 resampling method of Felsenstein (1985) was used to evaluate the stability of the resultant trees topologies.

### 2.3 Phenotypic characterization of strain MUM273b

The morphology and cultural characteristic of strain MUM273b were investigated on different media, including the ISP2, ISP3, ISP4, ISP5, ISP6, ISP7 (Shirling & Gottlieb, 1966), starch casein agar (SCA) (Küster & Williams, 1964), actinomycetes isolation agar (AIA) (Atlas, 2010), and nutrient agar (Mac Faddin, 1976), after incubation at 28°C for 14 days. The colony color of strain MUM273b was observed and compared with the ISCC‐NBS color charts (Kelly, 1964). Both light microscope (80i, Nikon) and scanning electron microscope (SEM) (TM‐1000, Hitachi) were used to observe the microscopic structures of the colony of strain MUM273b (7–14 days culture). Furthermore, the tolerance of strain MUM273b toward different temperature (4–40°C) and salinity (0–10% (w/v) of NaCl) was evaluated for 14 days on ISP2 agar. Strain MUM273b was also grown in TSB with adjusted pH to check its pH tolerance from 2.0 to 10.0. The ability to produce melanoid pigments of strain MUM273b was assessed on ISP7 agar (Lee, Zainal, Azman, Eng, Ab Mutalib, et al., 2014; Lee, Zainal, Azman, Eng, Goh, et al., 2014). Hemolytic activity of strain MUM273b was tested on blood agar (Carrillo, Mardaraz, Pitta‐Alvarez, & Giulietti, 1996). A range of enzymatic tests (catalase, amylase, cellulase, chitinase, lipase, protease, and xylanase) was performed to evaluate the enzyme productivity of strain MUM273b using ISP2 (Lee, Zainal, Azman, Eng, Ab Mutalib, et al., 2014; Lee, Zainal, Azman, Eng, Goh, et al., 2014; Meena, Rajan, Vinithkumar, & Kirubagaran, 2013). Antibiotic susceptibility of strain MUM273b was determined by the disc diffusion method (Shieh, Chen, Chaw, & Chiu, 2003). The carbon and nitrogen source utilization and chemical sensitivity of strain MUM273b were analyzed by Biolog GenIII MicroPlates (Biolog, USA).

### 2.4 Preparation of MUM273b extract

The fermentation process was conducted by inoculating a 14 days broth culture of strain MUM273b into an Erlenmeyer flask containing the Han's Fermentation Media 1 (Biomerge, Malaysia) (Hong et al., 2009; Lee et al., 2012). The inoculated media were fermented in rotary shaker at 200 rpm for 10 days at 28°C. The biomass was separated from the supernatant by centrifugation at 12,000×*g* for 15 min. The supernatant was filtered using filter paper (Whatman, UK) and freeze‐dried. The dried product was subject to methanol extraction for 72 hr. Subsequently, the organic solvent was collected by filtration prior to rotary evaporation at 40°C. After total removal of the organic solvent, the product was weighed and dissolved in dimethyl sulfoxide (DMSO).

### 2.5 Antioxidant activities of MUM273b extract

#### 2.5.1 DPPH‐radical scavenging activity

DPPH (2,2‐diphenyl‐1‐picrylhydrazyl) radical scavenging activity of MUM273b extract was assessed as described in (Ser, Palanisamy, et al., 2015). MUM273b extract was reacted with 0.016% (w/v) DPPH in 95% (v/v) ethanol. For 20 min, the reaction was incubated in the dark at room temperature. The absorbance of the mixture was taken immediately at 515 nm with microplate reader. Gallic acid was the positive control. The following formula was used to calculate the DPPH radical scavenging activity of MUM273b extract:

$$\begin{array}{c} \%\;\text{DPPH scavenging activity}\\ \;=\frac{\text{Absorbance of control}-\text{Absorbance of sample}}{\text{Absorbance of control}}\times 100\% \end{array}$$

#### 2.5.2 ABTS radical scavenging activity

The 2,2′‐azino‐bis(3‐ethylbenzothiazoline‐6‐sulfonic acid) (ABTS) radical scavenging assay was performed as described in (Ser, Palanisamy, et al., 2016). ABTS radical cation (ABTS^·^) was generated by reacting ABTS stock solution at 7 mM with potassium persulfate at 2.45 mM for 24 hr. The ABTS radical solution was mixed with MUM273b extract preloaded in the 96‐well microplate at different concentrations. After 20 min incubation in the dark, the absorbance of the mixture was taken immediately at 734 nm with microplate reader. Gallic acid was the positive control. The following formula was used to compute the percentage ABTS scavenging activity of MUM273b extract:

$$\begin{array}{c} \%\;\text{ABTS scavenging activity}\\ \;=\frac{\text{Absorbance of control}-\text{Absorbance of sample}}{\text{Absorbance of control}}\times 100\% \end{array}$$

#### 2.5.3 Superoxide anion radical scavenging activity

The superoxide anion scavenging activity or superoxide dismutase (SOD)‐like activity of MUM273b extract was investigated following the manufacturer's protocol (19160 SOD Assay Kit‐WST, Sigma Aldrich). Briefly, MUM273b extract was mixed with respective reaction solutions accordingly before incubation at 37°C for 30 min. Absorbance of each reaction mixture was measured at 450nm using a microplate reader colorimetrically. The following formula was used to determine the superoxide anion scavenging activity or SOD‐like activity of MUM273b extract:

$$\begin{array}{c} \%\;\text{SOD like activity}\\ \;=\frac{\left(\text{Absorbance of control blank}-\text{Absorbance of buffer blank}\right)-\left(\text{Absorbance of sample}-\text{Absorbance of sample blank}\right)}{\text{Absorbance of control blank}-\text{Absorbance of buffer blank}}\times 100\% \end{array}$$

#### 2.5.4 Metal chelating activity

Metal chelating activity of MUM273b extract was measured as shown in previous studies (Adjimani & Asare, 2015; Dinis, Madeira, & Almeida, 1994). Briefly, FeSO4 at 2 mM was mixed with serially diluted MUM273b extract. Subsequently, ferrozine (5 mM) was added to start the reaction. After 10 min, absorbances of the mixtures were measured at wavelength of 562 nm. EDTA was the positive control. The following formula was used to calculate the metal chelating activity of MUM273b extract:

$$\begin{array}{c} \%\;\text{Metal chelating activity}\\ \;=\frac{\text{Absorbance of control}-\text{Absorbance of sample}}{\text{Absorbance of control}}\times 100\% \end{array}$$

### 2.6 Lipid peroxidation assay

To assess the inhibitory potential of MUM273b extract against lipid peroxidation, thiobarbituric acid reactive species (TBARS) assay was used to measure the malondialdehyde (MDA) formed from iron‐induced lipid peroxidation in lipid‐rich media, as described in Dasgupta and De (2004). In short, MUM273b extract was added into the egg homogenate prepared in phosphate‐buffered saline (PBS) (10% v/v). To induce lipid peroxidation, 100 μM of FeSO4 was added to the mixture. After 1 hr, ice‐cold 20% trichloroacetic acid was added to stop the reaction in 1 to 1 proportion. The MDA content in the supernatant was obtained by centrifugation at 1,200×*g* for 10 min and measured by using TBARS assay (Tan et al., 2017). The fluorescence intensity of the product was measured by fluorometer at 535 excitation/553nm emission. The following formula was used to calculate the inhibitory effect of MUM273b extract against lipid peroxidation (%):

$$\%\;\text{inhibition of lipid peroxidation}=\left(\frac{{\text{RFI}}_{\text{Blank}}-{\text{RFI}}_{\text{Sample}}}{{\text{RFI}}_{\text{Blank}}}\right)\times 100\%$$

RFI: relative fluorescence intensity; Blank: no extract added.

### 2.7 Total phenolic content (TPC) and total flavonoid content (TFC) estimation

The TPC of MUM273b extract was estimated using a 96‐microwell format of Folin‐Ciocalteu's (FC) reagent method (Zhang et al., 2006). Absorbance of the mixture in each well was measured at 750 nm. Meanwhile, the TFC of MUM273b extract was estimated by aluminum‐flavonoid complexes formation in 96‐microwell format (Herald, Gadgil, & Tilley, 2012). The absorbance was determined at 510 nm.

### 2.8 Cell culture

HaCaT human keratinocytes were cultured in DMEM containing 4.5 g/L glucose and l‐glutamine supplemented with 10% fetal bovine serum and 100× antibiotic/antimycotic (1 × 10^4^ units/ml penicillin, 10 mg/ml streptomycin and 25 μg/mL amphotericin B) (Gibco). The cells were maintained in a humid atmosphere of 5% CO_2_ at 37°C.

### 2.9 UVB irradiation

HaCaT human keratinocytes were seeded in 96‐well culture plates at density of 1x10^4^ cells/well and incubated in an atmosphere of 5% CO_2_ at 37°C overnight for attachment. Prior to UVB irradiation, culture medium was replaced with a thin layer of PBS in the presence or absence of MUM273b extract in a series of concentrations. The cells were irradiated with Philip UVB Broadband TL 20W/12 phototherapy lamp (Philip, Amsterdam) with a wavelength range between 290 and 315 nm. The intensity of irradiation was 50 mJ/cm^2 ^(Mahendra et al., 2019). UV intensity was measured using a UV light meter UV‐340A (Lutron, USA). After UVB irradiation, the PBS was replaced with fresh growth medium and incubated in 5% CO_2_ at 37°C for 24 hr before subjected to MTT viability assay.

### 2.10 MTT viability assay

The viability of HaCaT human keratinocytes in the 96‐well plate was measured using 3‐(4,5‐dimethylthiazol‐2‐yl)‐2,5‐diphenyltetrazolium bromide (MTT) assay (Goh & Kadir, 2011). The assay was performed by adding 20 µl of MTT solution (5mg/ml) to each well and incubated at 37°C with 5% CO_2_ for 4 hr. The medium was discarded by gentle aspiration. The formazan crystals were dissolved in 100 µl of DMSO and the absorbance of each well was measured spectrophotometrically at 570 nm (with 650 nm as reference wavelength).

### 2.11 Phase‐contrast microscopy

The effect of UVB radiation and the UVB‐protective effect of MUM273b extract on HaCaT human keratinocytes were examined morphologically with an inverted light microscope.

### 2.12 Gas chromatography–mass spectrometry analysis

Gas chromatography–mass spectrometry analysis (GC‐MS) was performed to profile the possible bioactive compounds present in MUM273b extract (Supriady, Kamarudin, Chan, Goh, & Kadir, 2015). The detection involved the use of the Agilent Technologies 6980N (GC) equipped with 5979 Mass Selective Detector (MS), HP‐5MS (5% phenyl methyl siloxane) capillary column of dimensions 30.0 m × 250 µm × 0.25 µm and helium as carrier gas at 1 ml/min. For the initial 10 min, the column temperature was maintained at 40°C. The run was initiated with increasing temperature of 3°C/min until 250°C and followed by maintaining the temperature isothermally for 5 min. The MS was operating at 70 eV. By comparing to the mass spectral data available in W9N11 MS library, the detected compounds in MUM273b extract were identified.

### 2.13 Statistical analysis

All the antioxidant tests were conducted in quadruplicates. Statistical analysis was performed using SPSS software. The significant difference between the treated and untreated groups was determined by one‐way analysis of variance (ANOVA) and Tukey's post hoc analysis. A difference was considered statistically significant when *p* ≤ 0.05. The relationship between the total phenolic content and the antioxidant capacity of the extract was evaluated using Pearson's correlation analysis.

## 3 RESULTS

### 3.1 Identification of strain MUM273b by 16S rRNA phylogenetic analysis

The sequencing result yielded a 1,406 bp 16S rRNA gene of strain MUM273b. The gene has been deposited in the database (GenBank accession number MK611768). Based on the blast result of Ezbiocloud server, 16S rRNA gene sequence of strain MUM273b showed high sequence similarity to *S. albogriseolus* NRRLB‐1305^T^ (98.03%), *S. viridodiastaticus* NBRC13106^T ^(98.03%) and *S. longispororuber* NBRC13488^T^ (98.03%). The phylogenetic tree indicated that strain MUM273b clustered within the genus *Streptomyces*, forming a clade with *S. coeruleorubidus* ISP5145^T^, *S. albogriseolus* NRRLB‐1305^T^, and *S. viridodiastaticus* NBRC13106^T^ at a bootstrap value of 93% (Figure 1).

### 3.2 Phenotypic characteristics of strain MUM273b

**Figure 2:**
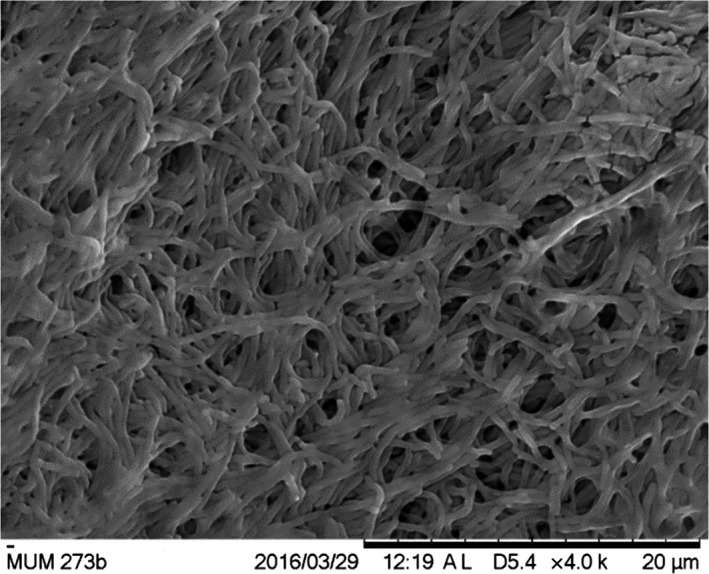
The scanning electron micrographs of *Streptomyces* sp. MUM273b. It appears as smooth filaments and branch to form a network of filaments called mycelium

Strain MUM273b is Gram‐positive and aerobic. The aerial and substrate mycelium of strain MUM273b appears in different colors on different agar. To illustrate, a yellow color substrate mycelium and pale‐yellow aerial mycelium could be observed when grown on ISP2 agar. At 28°C, growth of strain MUM273b was observed on ISP6, ISP7, AIA, and NA agar, but weaker growth was shown on ISP2, ISP3, ISP5, and SCA. However, strain MUM273b does not grow on ISP4 agar. Moreover, smooth and dense network of filaments could be visible when viewing strain MUM273b under SEM (Figure 2). These morphological characteristics further indicate that strain MUM273b belongs to the genus *Streptomyces*.

In addition, strain MUM273b does not grow at a temperature greater than 40°C, with optimal growth at 32°C. It prefers to grow at pH7 while growth can be seen under pH in the range 6–8. It has NaCl tolerance up to 8% (w/v) (optimum at 2% (w/v)). Strain MUM273b is positive for catalase while negative for hemolytic activity. It does not produce melanoid pigment. It can produce amylase and cellulase enzymes, but not lipase, xylanase, protease, and chitinase. From the Biolog assay, strain MUM273b was shown to be positive for wide variety of carbon and nitrogen sources utilization (Table 1). Moreover, strain MUM273b was shown to be chemically resistant to 1% sodium lactate and rifamycin RV. Besides that, strain MUM273b was shown to be susceptible to chloramphenicol, erythromycin, gentamicin, tetracycline, and vancomycin. Meanwhile, it exhibited resistance toward antibiotic such as cefotaxime, Penicillin G, ampicillin, ampicillin sulbactam, and nalidixic acid.

**Table 1:** The utilization of carbon and nitrogen sources by *Streptomyces* sp. MUM273b strain

Carbon and nitrogen utilization			
3‐Methyl glucose	−	d‐Turanose	+
Acetic acid	+	Formic acid	−
Acetoacetic acid	+	Gelatin	+
α‐d‐Glucose	+	Gentiobiose	+
α‐d‐Lactose	+	Glucuronamide	+
α‐Hydroxy‐butyric acid	+	Glycerol	+
α‐Keto‐butyric acid	+	Glycyl‐l‐proline	+
α‐Keto‐glutaric acid	+	Inosine	+
β‐Hydroxy‐d,l‐butyric acid	+	l‐Alanine	+
β‐Methyl‐d‐glucoside	+	l‐Arginine	+
Bromo‐succinic acid	+	l‐Aspartic acid	+
Citric acid	+	l‐Fucose	−
d‐Arabitol	+	l‐Galactonic acid lactone	+
d‐Aspartic acid	−	l‐Glutamic acid	+
d‐Cellobiose	+	l‐Histidine	+
Dextrin	+	l‐Lactic acid	+
d‐Fructose	+	l‐Malic acid	+
d‐Fructose‐6‐phosphate	+	l‐Pyroglutamic acid	+
d‐Fucose	−	l‐Rhamnose	+
d‐Galactose	+	l‐Serine	+
d‐Galacturonic acid	+	Methyl pyruvate	+
d‐Gluconic acid	+	Mucic acid	+
d‐Glucose‐6‐phosphate	+	Myo‐inositol	+
d‐Glucuronic acid	+	N‐acetyl‐b‐d‐mannosamine	−
d‐Lactic acid methyl ester	+	N‐acetyl‐d‐galactosamine	−
d‐Malic acid	+	N‐acetyl‐d‐glucosamine	+
d‐Maltose	+	N‐acetyl‐neuraminic acid	−
d‐Mannitol	+	Pectin	+
d‐Mannose	+	ρ‐Hydroxy‐phenylacetic acid	−
d‐Melibiose	+	Propionic acid	+
d‐Raffinose	+	Quinic acid	+
d‐Saccharic acid	+	Stachyose	−
d‐Salicin	+	Sucrose	−
d‐Serine	−	Tween 40	+
d‐Sorbitol	+	γ‐Amino‐butyric acid	+
d‐Trehalose	+		

Note

“+” indicates positive utilization; ‘−’ indicates negative utilization.

### 3.3 Antioxidant activities

Several antioxidant activity assays were performed to assess the possible different mechanisms of MUM273b extract in exerting antioxidant activities. Table 2 tabulates the results of the antioxidant activities of MUM273b extract. The MUM273b extract was shown to exhibit significant DPPH scavenging activity of 5.00 ± 1.03% to 8.83 ± 0.87% at 2–4 mg/ml, suggesting the potential of MUM273b extract to donate hydrogen atom to the DPPH radical. The MUM273b extract was also shown to scavenge ABTS^·+^ radical formed through the reaction between ABTS and potassium persulfate. The results showed that MUM273b extract possessed significant ABTS radical scavenging activity (*p* < 0.05) measured from 7.08 ± 1.79% to 32.26 ± 0.61% at tested concentrations from 0.25 mg/ml to 4 mg/ml.

**Table 2:** The antioxidant activities demonstrated by Streptomyces MUM273b extract in different antioxidant assays

Concentration of *Streptomyces* sp. MUM273b extract (μg/ml)	Antioxidant activities			
	DPPH radical scavenging activity (%)	ABTS radical scavenging activity (%)	Superoxide dismutase‐like activity (%)	Metal‐chelating activity (%)
250	ND	7.08 ± 1.79*	ND	11.08 ± 1.40*
500	ND	10.03 ± 2.16*	1.76 ± 2.66	11.56 ± 1.74*
1,000	1.76 ± 0.31	12.31 ± 1.09*	7.07 ± 1.75*	14.18 ± 2.8*
2000	5.00 ± 1.03*	16.15 ± 2.73*	13.89 ± 1.91*	17.90 ± 2.68*
4,000	8.83 ± 0.87*	32.26 ± 0.61*	22.47 ± 2.73*	23.44 ± 1.63*
Gallic acid				
6.25	34.48 ± 3.42*	56.24 ± 4.89*	—	—
Catechin				
7.5	—	—	66.65 ± 0.88*	—
EDTA				
15.6	—	—	—	19.77 ± 3.31*

Abbreviation: ND, not detected.

* —Statistically significance (*p* < 0.05) when compared to control (without extract).

Superoxide anion radical (O_2_^·−^) represents a major primary ROS that can further interact with other molecules to generate several different oxygen metabolites ROS, thus it is crucial to impede this process to prevent further generation of harmful ROS such as the hydroxyl radical (Bergamini, Gambetti, Dondi, & Cervellati, 2004). In this study, a superoxide detector, WST‐1 was used to measure the SOD‐like activity of MUM273b extract. The assay showed that MUM273b extract was capable of scavenging O_2_^·−^ as shown by the reduced intensity of yellow water‐soluble WST formazan formed from reduction of WST‐1 by O_2_^·−^. The assay revealed that MUM273b extract exhibited significant SOD‐like activity of 7.07 ± 1.75% to 22.47 ± 2.73% at concentrations ranging from 1 to 4 mg/ml.

To show that MUM273b extract exhibits metal‐chelating activity, the inhibition of purple complex forming from the reaction of Fe^2+ ^ion and ferrozine by MUM273b was examined. The result showed that MUM273b extract exhibits significant metal chelating activity measured from 11.08 ± 1.40% to 23.44 ± 1.63% at concentrations ranging from 0.25 to 4 mg/ml.

**Figure 3:**
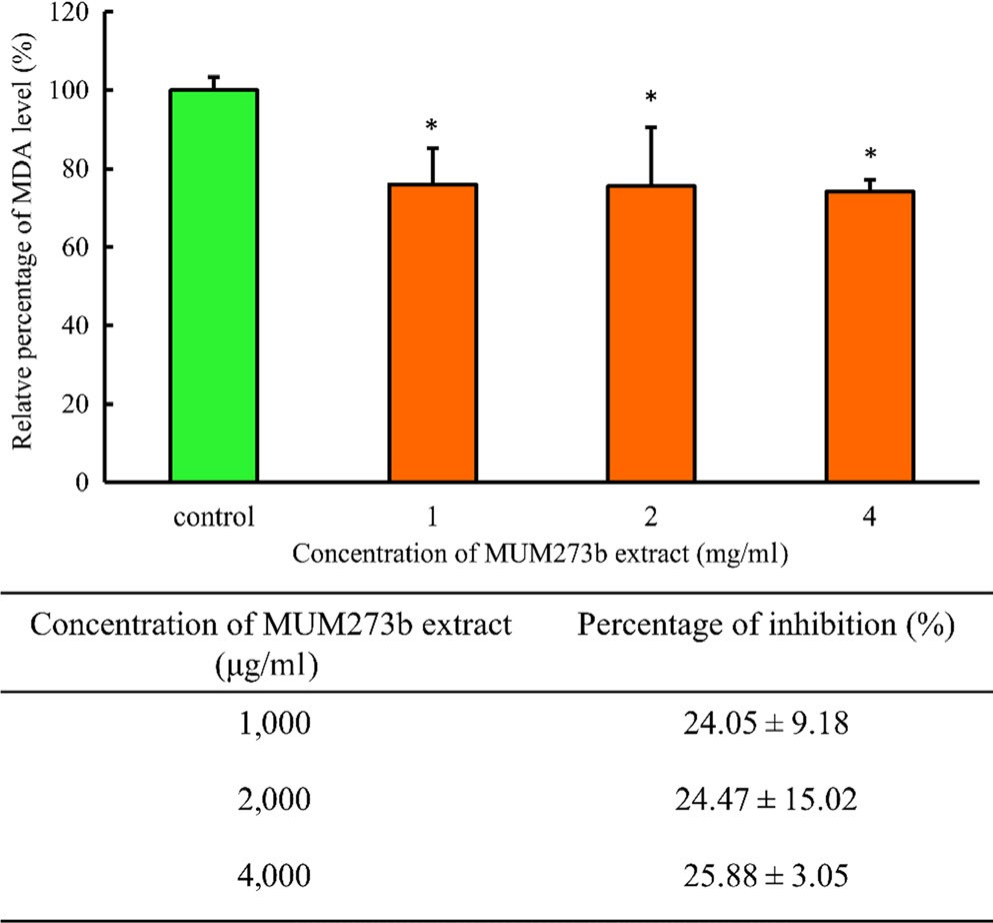
Effect of MUM273b extract against lipid peroxidation induced by Fe^2+^. MDA level was quantified using TBARS assay. All data are presented as mean ± *SD* (*n* = 3). *indicates *p < *0.05 between control (without extract) and MUM273b extract added samples. MDA, malondialdehyde; TBARS, thiobarbituric acid reactive species

On top of that, the inhibitory effect of MUM273b extract on iron‐induced peroxidation in egg yolk homogenate was evaluated. MUM273b extract was demonstrated to suppress the generation of MDA as a result of adding Fe^2+^ into the egg homogenate. The generation of MDA was shown to be reduced significantly when treated with MUM273b extract as compared to the control group (Figure 3). At 4 mg/ml, MUM273b extract exhibited 25.88 ± 3.05% of inhibition against Fe^2+^‐induced lipid peroxidation.

### 3.4 Estimation of TPC and TFC of MUM273b extract

The FC reagent method was used to detect the presence of phenolic compounds in MUM273b extract by estimating the total concentration of phenolic hydroxyl group. The FC reagent reacts with the phenolic hydroxyl group to form blue complex with maximum absorbance at 750 nm (Singleton, Orthofer, & Lamuela‐Raventós, 1999). The assay suggested the presence of phenolic compounds in MUM273b extract was evident by the increasing absorbance of the blue complex with the increasing concentration tested. Meanwhile, MUM273b extract was shown to be negative for flavonoids. Moreover, Pearson's correlation analysis indicated the strongest positive correlation between the TPC and SOD‐like activity of MUM273b extract with *r* = 0.997 (*p* < 0.05) (Table 3). The positive correlation suggested that phenolic compounds may be the main contributor to the radical scavenging activities exhibited by MUM273b extract.

**Table 3:** Pearson's correlation coefficients between TPC and antioxidant activities of *Streptomyces* MUM273b extract

Antioxidant activities	Phenolic content
DPPH radical scavenging activity	*r* = 0.977*
ABTS radical scavenging activity	*r* = 0.916*
SOD‐like activity	*r* = 0.997*
Metal‐chelating activity	*r* = 0.973*

Abbreviation: SOD, superoxide dismutase.

* Correlation is significant at the 0.05 level.

### 3.5 Inhibition of UVB‐induced keratinocyte death

**Figure 4:**
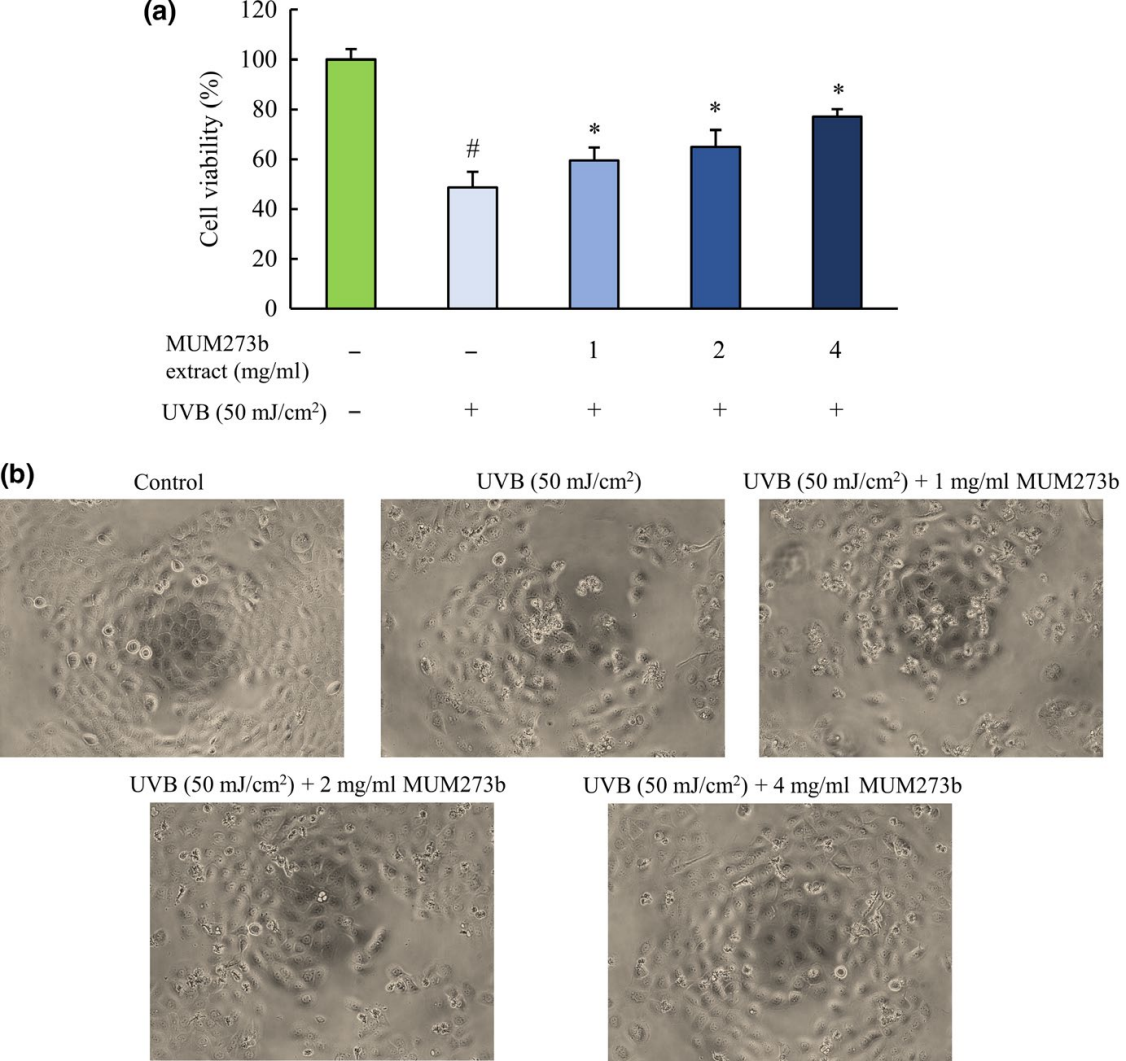
Protective effect of MUM273b extract against UVB‐induced cytotoxicity in HaCaT keratinocytes. (a) The HaCaT cells were exposed to UVB (50 mJ/cm^2^) in the presence of MUM273b extract at different concentrations. The cell viability was measured by MTT assay after 24 hr. All data are present as mean ± *SD* (*n* = 5). # indicates *p < *0.05 between control (without UVB) and cells exposed to UVB (50 mJ/cm^2^). *indicates *p < *0.05 between cells (without extract) and MUM273b extract‐treated cells after UVB exposure. (b) The morphological changes of HaCaT cells observed under phase‐contrast microscopy (×100)

To investigate the cytoprotective effect of MUM273b extract against UVB‐induced cytotoxicity, HaCaT keratinocytes were exposed to UVB (50 mJ/cm^2^) in the presence of different concentrations of MUM273b extract (1, 2 and 4 mg/ml) prior to MTT assay (Mahendra et al., 2019). As shown in Figure 4a, the cell viability of HaCaT cells was reduced significantly after exposure to UVB at the intensity of 50 mJ/cm^2^. Meanwhile, the MUM273b extract was shown to inhibit UVB‐induced keratinocyte cell death at concentrations of 1, 2, and 4 mg/ml. Furthermore, the morphological changes in HaCaT induced by UVB were also reversed by MUM273b extract (Figure 4b).

### 3.6 Detection of bioactive compounds in MUM273b extract

**Figure 5:**
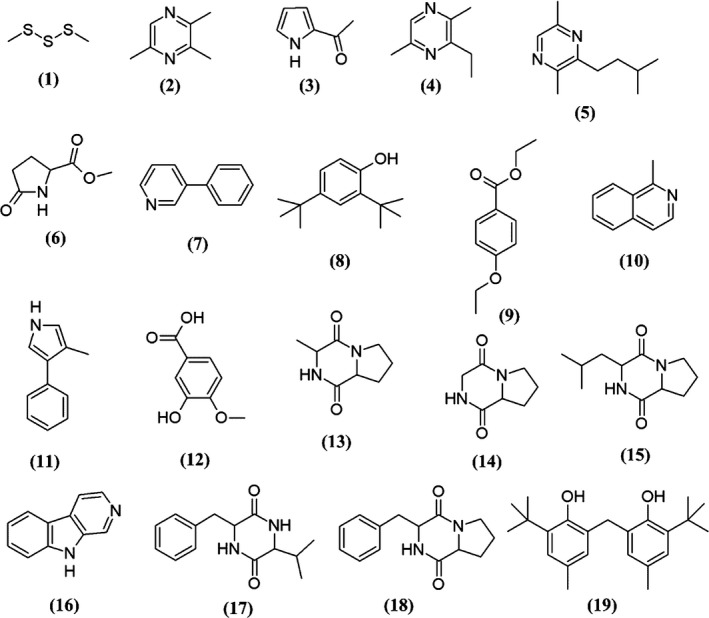
Chemical structures of constituents detected in MUM273b extract

The results of GC‐MS analysis revealed that MUM273b extract contains several groups of chemical compounds, including the pyrrole, pyrazine, ester, phenols, and cyclic dipeptides. The potential bioactive compounds present in MUM273b extract were identified by referring to the comparison between their mass spectra to standard mass spectra available in the database (W9N11 MS library). Table 4 lists the chemical compounds detected in term of their retention time, molecular weight, and molecular formula. Figure 5 depicts the chemical structures detected in MUM273b extract.

**Table 4:** Chemical constituents detected in of *Streptomyces* sp. MUM273b extract

No.	Constituents [synonym]	Retention time (min)	Molecular formula	Molecular Weight (MW)	Similarity (%)
1	Trisulfide, dimethyl	17.57	C_2_H_6_S_3_	126	91
2	Pyrazine, trimethyl‐	19.698	C_7_H_10_N_2_	122	78
3	2‐Acetylpyrrole	23.446	C_6_H_7_NO	109	87
4	Pyrazine, 3‐ethyl‐2,5‐dimethyl‐	24.332	C_8_H_12_N_2_	136	90
5	Pyrazine, 2,5‐dimethyl‐3‐(3‐methylbutyl)	36.091	C_11_H_18_N_2_	178	80
6	dl‐Proline, 5‐oxo‐methyl ester	38.929	C_6_H_9_NO_3_	143	80
7	Pyridine, 3‐phenyl‐	42.139	C_11_H_9_N	155	81
8	Phenol, 2,4‐bis(1,1‐dimethylethyl)‐	44.474	C_14_H_22_O	206	96
9	Benzoic acid, 4‐ethoxy‐,ethyl ester	44.897	C_11_H_14_O_3_	194	96
10	Isoquinoline, 1‐methyl‐	45.618	C_10_H_9_N	143	91
11	3‐Methyl‐4‐phenyl‐1H‐pyrrole	46.991	C_11_H_11_N	157	95
12	3‐Hydroxy‐4‐methoxybenzoic acid	51.918	C_8_H_8_O_4_	168	86
13	(3R,8aS)‐3‐methyl‐1,2,3,4,6,7,8,8a‐octahydropyrrolo[1,2a]pyrazine‐1,4‐dione [Cyclo(Pro‐Ala)]	52.742	C_8_H_12_N_2_O_2_	168	90
14	Pyrrolo[1,2a]pyrazine‐1,4‐dione, hexahydro [Cyclo(Gly‐Pro)]	53.738	C_7_H_10_N_2_O_2_	154	96
15	1,4‐Diaza‐2,5‐dioxo‐3‐isobutyl bicyclo[4.3.0]nonane [Cyclo(Leu‐Pro)]	59.311	C_11_H_18_N_2_O_2_	210	78
16	9H‐Pyrido[3,4‐b]indole	60.53	C_11_H_8_N_2_	168	95
17	2,5‐Piperazinedione, 3‐(1‐methylethyl)‐6‐(phenylmethyl)‐ [Cyclo(Phe‐Val)]	69.004	C_14_H_18_N_2_O_2_	246	90
18	3‐Benzyl‐1,4‐diaza‐2,5‐dioxobicyclo[4.3.0]nonane [Cyclo(Pro‐Phe)]	72.18	C_14_H_16_N_2_O_2_	244	99
19	Phenol,2,2′‐methylenebis[6‐(1,1‐dimethylethyl)‐4‐methyl‐	73.518	C_23_H_32_O_2_	340	97

## 4 DISCUSSION

The continuous need for research on natural products is required to keep pace with ever‐growing demands for better therapeutics. Mangrove‐associated microbes continue to be the highlight for natural product research. Microorganisms that inhabit within the intertidal region are believed to have adapted specific metabolic pathways that aid in resisting the natural stressors such as constant changes of tidal gradient, temperature, and high salinity (McKee, 1995; Xu, 2015) for survival, subsequently leading to synthesis of unique and interesting secondary metabolites (Hong et al., 2009). Therefore, mangrove environment is becoming the focus of considerable microbiological interest, especially in the effort to discover novel microbial natural product.

Given the growing number of novel bacteria discoveries from mangrove ecosystem, increasing attention has also been placed on the exploration of novel or unusual biomolecules from the mangrove‐associated microbes (Xu, 2015). As one of the largest microbial community in the mangrove ecosystem, *Streptomyces* sp. is known to be valuable and rich bioresources for bioactive compounds, to which numerous have been approved as clinically used drugs (Patridge, Gareiss, Kinch, & Hoyer, 2016; Vilhena & Bettencourt, 2012). Thus, this study aimed to investigate the bioactive potentials of the *Streptomyces* sp. derived from mangrove environment. This study has successfully isolated a *Streptomyces* sp., strain MUM273b from a soil sample collected from mangrove forest in Selangor, Malaysia. Furthermore, the mangrove‐associated microbes are believed to have developed specific defense due to the constant exposure to the environmental stressors (Hong et al., 2009; Lee, Zainal, Azman, Eng, Ab Mutalib, et al., 2014; Lee, Zainal, Azman, Eng, Goh, et al., 2014; Tan et al., 2018). To overcome these environmental stressors, these microbes are required to produce unique metabolites for their survival (Hong et al., 2009). Exposure to solar UV radiation can result in numerous skin‐related conditions including, erythema, photoaging, and skin cancer. Given that the UVB triggers intracellular oxidative stress, many reports have revealed the use of antioxidant agents to protect cells from UVB‐induced damage (Salucci et al., 2014). Hence, this study further evaluated the antioxidative potential of strain MUM273b and its UVB ray protective properties.

This study has isolated strain MUM273b which is identified as *Streptomyces* sp. based on 16S rRNA gene phylogenetic analysis. The phylogenetic analysis showed that strain MUM273b shares 98.03% 16S rRNA gene sequence similarity with *S. albogriseolus* NRRLB‐1305^T^, *S. viridodiastaticus* NBRC13106^T ^(98.03%), and *S. longispororuber* NBRC13488^T^ (98.03%). Besides identifying strain MUM273b as genus *Streptomyces*, the phylogenetic analysis also suggested that strain MUM273b may have immense potential in biosynthesis of valuable secondary metabolites, thereby it was found to cluster with *S. albogriseolus* and *S. viridodiastaticus* which were previously reported to produce numerous bioactive metabolites (Li, Xu, Zhao, & Xu, 2010; Singh et al., 1994). To have a better understanding of the strain, we also performed phenotypic characterization of strain MUM273b including its morphological, physiological, and biochemical properties. The color of aerial mycelium is yellow and the substrate mycelium is pale‐yellow. Strain MUM273b was found to be capable of digesting both starch and CMC, suggesting that strain MUM273b may have the potential to be employed as industrial important strain for the production of essential enzymes such as amylase and cellulase. Furthermore, strain MUM273b demonstrates high salinity tolerance to 8% (w/v) NaCl and moderate temperature tolerance up to 40°C, as these characteristics are crucial to be equipped by mangrove‐associated microbes for their survival in the dynamic mangrove ecosystem.

The composition of substrates available during growth has great impact on the secondary metabolism of *Streptomyces* sp. (Ser, Law, et al., 2016). Thus, Biolog GEN III MicroPlate system was utilized to determine metabolic profile of strain MUM273b by determining the types of carbon and nitrogen utilization. The utilization assay showed that strain MUM273b was able to utilize a range of carbon and nitrogen sources, such as monosaccharides (α‐d‐glucose, d‐fructose, d‐galactose); disaccharides (d‐maltose, α‐d‐lactose, d‐trehalose but not sucrose); polysaccharides (pectin, gelatin and dextrin); glycoside (N‐acetyl‐d‐glucosamine), amino acids (l‐arginine, l‐histidine and l‐serine), and sugar alcohols (d‐mannitol and d‐sorbitol). Having these data, the process of medium optimization could be aided in future to improve the synthesis of desirable bioactive compounds at a larger scale.

Human skin cells or keratinocytes, as the major targets of UV, are constantly under oxidative stress induced by UVB irradiation via ROS generation, subsequently leading to cell death (Brash et al., 1991; Portugal, Barak, Ginsburg, & Kohen, 2007). Given that the ROS play pivotal roles in cell‐damaging oxidation process, cell or an organism's defense against of oxidative stress or attack by ROS could be prevented by antioxidants. Antioxidants function to protect cell or an organism from oxidative stress through neutralization of ROS which induces oxidative damages (Apak, Özyürek, Güçlü, & Çapanoğlu, 2016; Halliwell, 2011). The antioxidants function in several ways such as scavenging free radicals, interfering autoxidation chain reaction, converting the ROS into stable compounds, and chelating metal prooxidants (Devasagayam et al., 2004; Lobo, Patil, Phatak, & Chandra, 2010). The reason for being that the total antioxidant capacity of MUM273b extract was examined by several in vitro antioxidant assays. DPPH and ABTS assays are based on single electron transfer reaction (Prieto, Curran, Gowen, & Vázquez, 2015). This type of antioxidant assay evaluates the ability of a substance/extract to neutralize the radical indicators through different mechanisms such as electron transfer or hydrogen transfer. Generally, these rapid and simple assays serve as preliminary screening to assess the antioxidant potential of the extract. Given that the DPPH and ABTS radicals are not present in the biological systems, MUM273b extract was further tested for superoxide anion scavenging activity. The ability of MUM273b extract to scavenge O_2_^·− ^suggested that it may lower the O_2_^·− ^level as high O_2_^·−^ level has been associated to many pathological conditions such as cancer and cardiovascular diseases (Fukai, Folz, Landmesser, & Harrison, 2002; Lopez‐Lazaro, 2007; Pervaiz & Clement, 2007). Moreover, O_2_^·− ^is also the precursor of numerous reactive oxygen intermediates, including the highly reactive peroxynitrite molecule (ONOO^‐^) and hydroxide radical (^·^OH) (Bergamini et al., 2004). Therefore, the control of O_2_^·− ^generation is of great important with the aim to maintain the balance between the production rate of O_2_^·− ^and antioxidant capacity of the endogenous SOD enzymes defense system to protect from oxidative damages.

Iron is essential for all forms of life. However, excess iron can catalyze the Fenton reaction which involves the decomposition of hydrogen peroxide, leading to generation of ROS which damages lipids, proteins, and DNA (Prousek, 2007). Cutaneous damage has also been associated to iron‐catalyzed ROS generation (Kitazawa, Iwasaki, & Sakamoto, 2006). Thus, the ability of MUM273b extract in chelating metal is promising as it may reduce the increased catalytic iron level in skin resulted from the release of iron from ferritin upon exposure to UV radiation (Kitazawa et al., 2006). Furthermore, lipid peroxidation occurs when ROS damage cell membrane by peroxidation of fatty acids within the phospholipid membrane. The increased iron content in response to UV radiation could further accelerate lipid peroxidation, resulting in the production of mutagenic substances such as MDA (Halliwell & Chirico, 1993; Hazra, Biswas, & Mandal, 2008). This study demonstrated that MUM273b extract suppressed the iron‐induced peroxidation in egg yolk homogenate, suggesting that MUM273b extract could prevent the generation of by‐products of lipid peroxidation which can cause further damage to protein and DNA.

Based on these findings, MUM273b extract is shown to possess antioxidant activities that hold promise for the future development of antioxidant agents exerting multiple antioxidant mechanisms, including both free radical scavenging and metal‐chelating properties. These findings are line with previous studies on the isolation of antioxidants producing strains of *Streptomyces* species derived from the mangrove soil in Selangor, Malaysia (Tan et al., 2018, 2017). Among these strains of mangrove‐derived *Streptomyces* sp., the antioxidant activity demonstrated by the extract of strain MUM273b is found to be inferior to the previously reported *Streptomyces* extracts, in term of their radical scavenging and metal chelating activities (Tan et al., 2018, 2017). Meanwhile, a newly reported novel *Streptomyces* species, named as *Streptomyces monashensis* isolated from the East Malaysia mangrove soil, was demonstrated to produce metabolites exhibiting comparable ABTS radical scavenging activity with 12.33 ± 3.07% at 2 mg/ml (Law et al., 2019). Nevertheless, this study shows that MUM273b extract was capable of attenuating the UVB‐induced cytotoxicity in HaCaT cells and serves as the first report of *Streptomyces* strain isolated from Malaysia mangrove soil exhibiting UVB‐protective activity. Taken together, these findings suggested that the cytoprotective effect of MUM273b extract against UVB irradiation might be due to its antioxidant properties, including scavenging free radicals and inhibiting lipid peroxidation.

Phenolic compounds have been well recognized for their antioxidant properties (Martins, Barros, & Ferreira, 2016; Shahidi & Ambigaipalan, 2015). The strong correlation between the tested antioxidant capacity and the total phenolic content suggested the presence of phenolic compounds in MUM273b extract and attributed to its antioxidant properties. Phenolic antioxidants have been known to interfere with oxidation process, mediating through free radical terminators and also metal chelators (Zamora & Hidalgo, 2016). Studies evidenced that phenolic compounds are effective in exerting protective effect against pathological conditions such as cancer and cardiovascular disease (Martins et al., 2016). Nevertheless, MUM273b extract was subjected to GC‐MS analysis to profile the chemical compounds which may confer the antioxidant capability of the extract.

Based on the GC‐MS results, MUM273b extract was shown to contain compounds from different chemical groups such as sulfur‐containing compound, pyrrole, pyrazine, esters, phenolics, and cyclic dipeptides. In line with several previous studies, these groups of chemicals were also reported in the bacterial fermentation broth or extracts derived from Actinomycetes and *Streptomyces* sp. For instance, dimethyltrisulfide (**1**) (Groenhagen, Maczka, Dickschat, & Schulz, 2014), trimethylpyrazine (**2**) (Citron, Barra, Wink, & Dickschat, 2015), 2‐acetylpyrrole (**3**) (Morgenstern, Paetz, Behrend, & Spiteller, 2015), pyrazine,2,5‐dimethyl‐3‐(3‐methylbutyl) (**4**) (Citron et al., 2015), phenol,2,4‐bis(1,1‐dimethylethyl)‐ (**8**) (Kumar, Duraipandiyan, & Ignacimuthu, 2014), benzoic acid,4‐ethoxy‐,ethyl ester (**9**) (Sharma, Goel, Dureja, & Uniyal, 2010), (3R,8aS)‐3‐methyl‐1,2,3,4,6,7,8,8a‐octahydropyrrolo[1,2a]pyrazine‐1,4‐dione (**13**) (Tan et al., 2017), pyrrolo[1,2a]pyrazine‐1,4‐dione, hexahydro (**14**) (Ser, Palanisamy, et al., 2015), 1,4‐diaza‐2,5‐dioxo‐3‐isobutyl bicyclo[4.3.0]nonane (**15**) (Ser, Ab Mutalib, et al., 2015), 9H‐Pyrido[3,4‐b]indole (**16**) (Tan, Ser, et al., 2015), 2,5‐piperazinedione, 3‐(1‐methylethyl)‐6‐(phenylmethyl)‐ (**17**) (Alshaibani et al., 2017), 3‐benzyl‐1,4‐diaza‐2,5‐dioxobicyclo[4.3.0]nonane (**18**) (Jog, Pandya, Nareshkumar, & Rajkumar, 2014; Sharma, Kalita, & Thakur, 2016) and phenol,2,2′‐methylenebis[6‐(1,1‐dimethylethyl)‐4‐methyl‐ (**19**) (Tan, Ser, et al., 2015).

Phenol,2,4‐bis(1,1‐dimethylethyl)‐ (**8**), 3‐hydroxy‐4‐methoxybenzoic acid (**12**) and phenol, 2,2′‐methylenebis[6‐(1,1‐dimethylethyl)‐4‐methyl‐ (**19**) were the phenolic compounds detected in the MUM273b extract. In line with the results of total phenolic content, the GC‐MS analysis further supported the evidence of phenolic compounds present in MUM273b extract. Previously, *Streptomyces* fermentation broth/extract was also reported to contain phenolic compounds (Ser, Ab Mutalib, et al., 2015; Ser, Palanisamy, et al., 2016). Phenolic compounds are well known antioxidants which exert their antioxidant effects by scavenging free radicals, donating atoms or electron, or chelating metal cations (Sulaiman et al., 2011; Yogeswari, Ramalakshmi, Neelavathy, & Muthumary, 2012). Therefore, the antioxidant capacity of MUM273b extract could be conferred by the phenolic compounds.

Besides that, cyclic dipeptide or 2,5‐diketopiperazines (DKP) were another group of the chemical compounds detected in MUM273b extract. DKP constitutes of simplest peptide derivatives which are commonly found in nature (Prasad, 1995). The cyclic dipeptides present in MUM273b extract are (3R,8aS)‐3‐methyl‐1,2,3,4,6,7,8,8a‐octahydropyrrolo[1,2a]pyrazine‐1,4‐dione (**13**), pyrrolo[1,2a]pyrazine‐1,4‐dione, hexahydro (**14**), 1,4‐diaza‐2,5‐dioxo‐3‐isobutyl bicyclo[4.3.0]nonane (**15**), 2,5‐piperazinedione, 3‐(1‐methylethyl)‐6‐(phenylmethyl)‐ (**17**), 3‐benzyl‐1,4‐diaza‐2,5‐dioxobicyclo[4.3.0]nonane (**18**). A number of studies have also reported the detection of these cyclic dipeptides in the fermentation broth of microbes (Ser, Palanisamy, et al., 2016; Vazquez‐Rivera et al., 2015; Würth, Barbieri, & Florio, 2014). These cyclic dipeptides were shown to exhibit antioxidant activities (Ser, Palanisamy, et al., 2015; Tan, Ser, et al., 2015). Besides that, a recent study by Maciel, Tavares, Caluz, Gaspar, and Debonsi (2018) demonstrated the isolation of compounds under the group of 2,5‐diketopiperazines from the metabolites of mangrove‐derived endophytic fungus showing UVB‐protective properties. Given that the detected chemical compounds were previously reported to exhibit antioxidant and potentially UV‐protective properties, the antioxidant and UV‐protective effects of *Streptomyces* MUM273b extract may be contributed by these chemical compounds. Taken together, this work has further given credit to the current knowledge of the bioactive potential of *Streptomyces* bacteria that live in mangrove environment, possibly becoming the next great contributor to the research of antioxidative agents aiming for protection against UVB‐induced skin diseases.

## 5 CONCLUSION

In summary, the research work isolated a *Streptomyces* strain derived from mangrove soil in Malaysia, which exhibits antioxidants producing abilities. The extract of the fermented broth of *Streptomyces* strain MUM273b exhibits DPPH, ABTS, and superoxide anion radicals scavenging activities. MUM273b extract was also capable of chelating metal ion and inhibiting iron‐induced lipid peroxidation. MUM273b extract also protected keratinocytes against UVB‐induced cytotoxicity. The presence of bioactive constituents including phenolic compounds and cyclic dipeptides may be responsible for the antioxidant and UVB‐protective activities of MUM273b extract. As a whole, the results of this study highlighted that the mangrove‐derived *Streptomyces* in particular strain MUM273b possess immense potential to synthesize antioxidative and UVB‐protective metabolites and hence could be exploited for future development as a functional ingredient in cosmeceutical applications.

## CONFLICT OF INTERESTS

The authors declare no conflict of interest.

## AUTHOR CONTRIBUTIONS

LT‐HT, K‐MC, L‐HL, and B‐HG performed the experiments and data analysis as well the manuscript writing. Technical supports and proofreading were contributed by B‐HG, L‐HL, T‐MK, Y‐YY, and K‐GC. L‐HL, K‐GC, and B‐HG also contributed to the funding of the project. L‐HL and B‐HG founded the research project.

## ETHICS STATEMENT

None required.

## Data Availability

All data are provided in full in the results section of this paper apart from the 16S rRNA gene sequence of strain MUM273b which is available at http://www.ncbi.nlm.nih.gov/genbank/ under accession number MK611768.
